# Impaired Vitamin D Metabolism in Hospitalized COVID-19 Patients

**DOI:** 10.3390/ph15080906

**Published:** 2022-07-22

**Authors:** Alexandra Povaliaeva, Viktor Bogdanov, Ekaterina Pigarova, Larisa Dzeranova, Nino Katamadze, Natalya Malysheva, Vitaliy Ioutsi, Larisa Nikankina, Liudmila Rozhinskaya, Natalia Mokrysheva

**Affiliations:** Endocrinology Research Centre, 117292 Moscow, Russia; bogdanov.viktor@endocrincentr.ru (V.B.); pigarova.ekaterina@endocrincentr.ru (E.P.); dzeranova.larisa@endocrincentr.ru (L.D.); nincho.1994@mail.ru (N.K.); malysheva.natalya@endocrincentr.ru (N.M.); ioutsi.vitalij@endocrincentr.ru (V.I.); nikankina.larisa@endocrincentr.ru (L.N.); rozhinskaya.ludmila@endocrincentr.ru (L.R.); mokrisheva.natalia@endocrincentr.ru (N.M.)

**Keywords:** COVID-19, SARS-CoV-2, vitamin D, vitamin D-binding protein

## Abstract

There is increasing data regarding the association between vitamin D and COVID-19. This study aimed to reveal the alterations of vitamin D metabolism in the setting of COVID-19. We examined 119 adult COVID-19 inpatients and 44 apparently healthy adult individuals with similar serum 25OH-D_3_ levels as a reference group. The assessment included serum biochemical parameters (total calcium, albumin, phosphorus, creatinine), parathyroid hormone (PTH), vitamin D-binding protein (DBP), vitamin D metabolites (25OH-D_3_, 25OH-D_2_, 1,25(OH)_2_D_3_, 3-epi-25OH-D_3_, 24,25(OH)_2_D_3_ and D_3_) and free 25OH-D. COVID-19 patients had in general very low vitamin D levels (median 25OH-D_3_ equals 10.8 ng/mL), accompanied by an increased production of the active vitamin D metabolite (1,25(OH)_2_D_3_), estimated as higher 1,25(OH)_2_D_3_ serum levels (61 [44; 81] vs. 40 [35; 50] pg/mL, *p* < 0.001) and lower 25OH-D_3_/1,25(OH)_2_D_3_ ratio (175 [112; 260] vs. 272 [200; 433], *p* < 0.001) which is presumably aimed at preventing hypocalcemia. Patients with COVID-19 also had elevated DBP (450 [386; 515] vs. 392 [311; 433] mg/L, *p* < 0.001) and low free 25OH-D levels (<LoB vs. 3.9 [3.2; 4.4] pg/mL, *p* < 0.001). Follow-up assessment of the COVID-19 inpatients showed recovery of the observed changes. Overall, hospitalized patients with an acute course of COVID-19 have not only very low levels of 25OH-D but also profound abnormalities in the metabolism of vitamin D regardless of the clinical course of the disease. These alterations might exacerbate existing vitamin D deficiency and its negative impact.

## 1. Introduction

COVID-19 pandemic remains a major worldwide public health threat. COVID-19 is an infectious disease with predominant lung involvement caused by a highly infectious novel coronavirus known as severe acute respiratory syndrome coronavirus-2 (SARS-CoV-2) [1]. Vitamin D has emerged as a factor that may be involved in susceptibility to the disease and is considered a potential therapeutic option.

**Figure 1:**
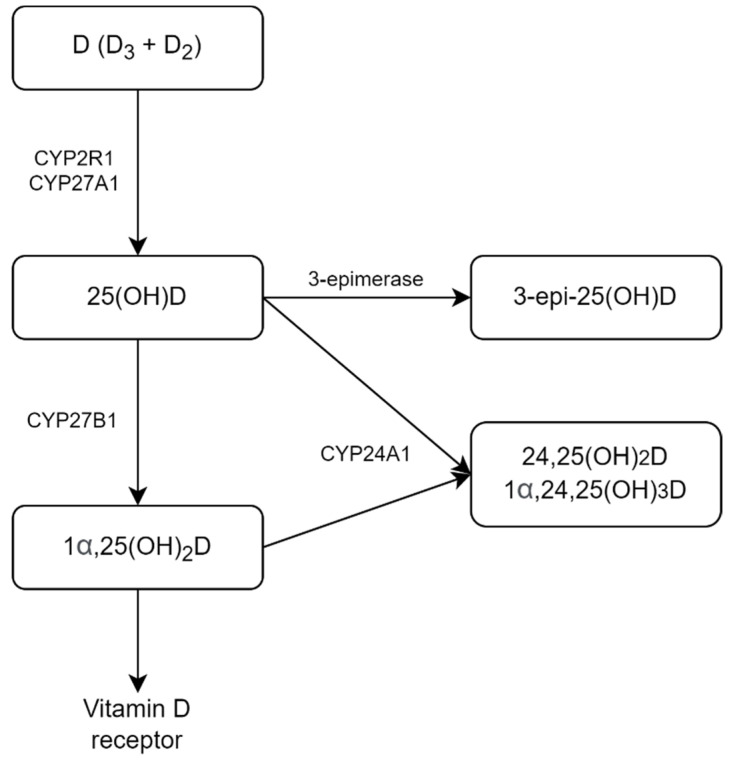
Scheme of vitamin D metabolism.

The three main steps in vitamin D metabolism, 25-hydroxylation, 1α-hydroxylation, and 24-hydroxylation, are all performed by cytochrome P450 mixed-function oxidases (CYPs) (Figure 1). To obtain biological activity, vitamin D undergoes two subsequent hydroxylation reactions—first to 25-hydroxyvitamin D (25OH-D), then to the active form 1,25-dihydroxyvitamin D (1,25(OH)_2_D). CYP2R1 is the most important 25-hydroxylase; CYP27B1 is the key 1-hydroxylase. Both 25OH-D and 1,25(OH)_2_D are catabolized by CYP24A1. Isomerization of the C-3 hydroxy group from the α to β orientation by 3-epimerase does not restrict the action of CYP27B1 or CYP24A1 but might reduce biologic effects [2].

Airway epithelia, alveolar macrophages, and dendritic cells all express 1α-hydroxylase and therefore have the potential to locally synthesize the active form of vitamin D (1,25(OH)_2_D) from its precursor (25OH-D) [3]. Although not demonstrated for coronaviruses such as SARS-CoV-2, for other viruses and other respiratory pathogens, activation of the innate immunity leading to increased local production of 1,25(OH)_2_D has been shown to enhance viral neutralization and clearance while modulating the subsequent proinflammatory response [4].

The effect of vitamin D on acquired immunity is considered predominantly in the context of its ability to influence the proliferation and phenotype of T cells [5]. In vitro 1,25(OH)_2_D modifies the cytokine profile of T-lymphocyte into an overall anti-inflammatory humoral immune response. Vitamin D may also stimulate the generation of the regulatory T cells, which are considered crucial for the induction of immune tolerance. These effects are suggestive of 1,25(OH)_2_D significance in preventing the cytokine storm associated with the severe respiratory disease caused by viral infections.

There is an increasing scientific interest in the possible association between vitamin D status and COVID-19 infection. According to the available evidence to date, most of the studies indicate a significant relationship between low 25OH-D levels and risk of SARS-CoV-2 infection [6,7,8,9,10,11,12,13], COVID-19 severity, and mortality [10,11,13,14,15], having considerable heterogeneity in methodological and statistical approach. The available evidence regarding the effectiveness of vitamin D in the treatment of COVID-19 comes predominantly from observational data and lacks thorough verification in clinical trials as the results of available meta-analyses are conflicting [16,17,18,19,20,21,22,23,24]. Vitamin D supplementation might be associated with improved clinical outcomes in COVID-19 patients, such as transfer to intensive care unit [16,19,20] and mortality rates [25]. Meta-analyses which included only randomized clinical trials and quasi-experimental studies showed uncertain or no evidence for the effectiveness of vitamin D supplementation in the treatment of COVID-19 [21,22]. However, vitamin D supplementation was significantly associated with reduced intensive care unit admission and mortality when administered after the diagnosis of COVID-19, according to the subgroup analysis [23]. One systematic review focused on patients aged 60 years or over demonstrated better primary clinical outcomes of COVID-19 in patients with vitamin D supplementation [24]. In a hypothesis-generating study by Giannini et al., comorbidity burden significantly modified the effect of vitamin D treatment with amplification of positive effect on clinical outcomes [26].

Overall, issues regarding the appropriate dose, duration, and mode of administration of vitamin D in COVID-19, as well as identifying the patient group with the greatest possible benefit, need further research. A comprehensive assessment of vitamin D metabolism during the course of COVID-19 could provide additional information for planning further clinical trials. Simultaneous measurement of 25OH-D and 24,25(OH)_2_D with the calculation of 25OH-D/24,25(OH)_2_D ratio has recently emerged as a valuable new diagnostic tool in the differential diagnosis of hypercalcemia particularly for screening of 24-hydroxylase deficiency [27,28,29,30,31]. Assessment of vitamin D metabolite profile and calculation of metabolites ratios is also a promising direction for better characterization of vitamin D status [29,32,33,34]; moreover, 25OH-D/24,25(OH)_2_D ratio has recently been shown to be associated with important clinical outcomes [35,36]. Tang et al. showed that the production of serum 1,25(OH)_2_D is favored over 24,25(OH)_2_D in vitamin D-insufficient young, healthy adults as the availability of vitamin D precursors in circulation diminishes [37]. A single retrospective study showed no association between parameters of vitamin D catabolism and fatal outcomes or the need for respiratory support in COVID-19 patients [38]. However, to the authors’ knowledge, there are no data regarding the activity of 1α-hydroxylase and production of active vitamin D metabolite in COVID-19, which might be of particular interest since promising clinical data was obtained with the administration of active vitamin D metabolites in COVID-19 patients [39].

This study aimed to reveal alterations in vitamin D metabolism in the setting of the COVID-19 course.

## 2. Results

COVID-19 group was presented by patients of various ages with a median age of 61 years, equally men and women (*p* > 0.05) (Table 1). Most of the patients with COVID-19 were overweight or obese; 17 patients (14%) had diabetes mellitus.

A detailed description of the condition on admittance and the course of inpatient treatment is presented in Table 2; most patients were characterized by a moderately severe course of infection. A total of 34 patients (29%) were characterized by a severe course of the disease, including 21 patients (18%) who were prescribed immunobiological treatment, 11 patients (9%) admitted or transferred to the intensive care unit, 10 patients (8%) with a fatal outcome.

The patients from the COVID-19 group had lower total calcium levels (*p* < 0.001) and serum albumin levels (*p* < 0.001), while levels of albumin-adjusted calcium were similar to the reference group (*p* = 0.06) (Table 3). Secondary hyperparathyroidism was revealed in 15 patients (13%) with COVID-19 and in 7 individuals (16%) from the reference group (*p* = 0.61), one COVID-19 patient had primary hyperparathyroidism. PTH levels were equal between the groups (*p* = 0.65). We also observed high creatinine levels in COVID-19 patients (*p* < 0.001), indicative of the decrease in kidney function and lower phosphorus levels (*p* = 0.04).

The detailed data on vitamin D metabolites, free 25OH-D, and DBP measurement in the COVID-19 group and the reference group with similar 25OH-D_3_ levels (*p* = 0.88) are presented in Table 4. Only 3 patients (3%) with COVID-19 had sufficient vitamin D levels, according to the Endocrine Society and the Russian Association of Endocrinologists guidelines (≥30 ng/mL [40,41]). COVID-19 patients had not only higher serum levels of 1,25(OH)_2_D_3_ (*p* < 0.001), but also lower 25OH-D_3_/1,25(OH)_2_D_3_ ratios (*p* < 0.001) implying increased production of the active metabolite (1,25(OH)_2_D_3_). They also had higher 25OH-D_3_/24,25(OH)_2_D_3_ ratios (*p* = 0.001). The rest of the studied metabolites did not differ significantly from the reference group. The levels of 25OH-D_2_ did not exceed 0.5 ng/mL in all examined individuals and detectable levels of vitamin D_3_ were observed only in 10 patients (8%) from the COVID-19 group (maximum 19.7 ng/mL) and in 7 individuals (16%) from the reference group (maximum 22.6 ng/mL). In addition, the COVID-19 group had significantly higher DBP levels (*p* < 0.001) and very low (mostly lower than the limit of blank) levels of free 25OH-D (*p* < 0.001) against the reference group.

**Figure 2:**
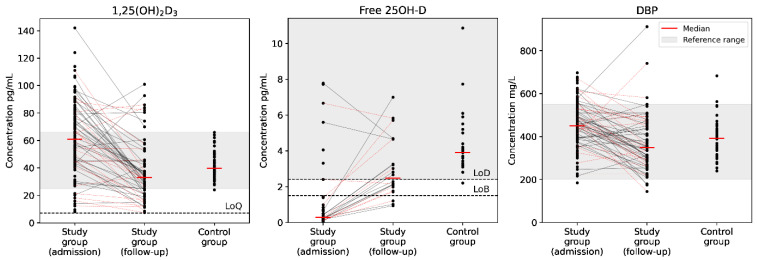
Dynamic evaluation of active vitamin D metabolite (1,25(OH)_2_D_3_), free 25OH-D, and vitamin D-binding protein (DBP) concentrations in patients with COVID-19 and comparison to the reference group. Data are shown as individual values. Limits of blank (LoB), detection (LoD), and quantitation (LoQ) are shown. Red dotted lines correspond to patients with a severe course of COVID-19.

Follow-up samples with median follow-up period 11 [9; 14] days were available in 62 patients (52%) from the COVID-19 group, of which 20 patients (32%) were characterized by severe course of the disease, defined as prescription of immunobiological treatment (n = 12), admission or transfer to the intensive care unit (n = 7) or fatal outcome (n = 5). We observed clinically insignificant change in 25OH-D_3_ levels (9.4 [5.2; 13.0] vs. 10.8 [6.2; 15.6] ng/mL, *p* < 0.001) and the corresponding change in 3-epi-25OH-D_3_ and 24,25(OH)_2_D_3_ levels (*p* < 0.001 for both parameters), however, there was no change in the 25OH-D_3_/24,25(OH)_2_D_3_ ratios (*p* = 0.07). At the same time, we observed prominent decrease in serum 1,25(OH)_2_D_3_ levels (*p* < 0.001) and concomitant increase in 25OH-D_3_/1,25(OH)_2_D_3_ ratios (*p* < 0.001), as well as significant changes in the levels of free 25OH-D (*p* = 0.002) and DBP (*p* < 0.001), approaching the levels characteristic of the reference group (Figure 2).

Patients with a severe course of COVID-19 showed no significant deviations in the studied laboratory parameters versus the main body of patients with a moderately severe course of the disease, except for a lower baseline total calcium (*p* = 0.04) (Table 5).

## 3. Discussion

First of all, our study further supported the prevalence of very low vitamin D levels in patients with COVID-19. The median level of 25OH-D_3_ in COVID-19 patients was only 10.8 ng/mL, which corresponds to a pronounced vitamin D deficiency in a major part of the examined patients [41] and depicts worse vitamin D status than epidemiological data from a similar season [44]. Similar results have been demonstrated in a number of previous studies from various countries [45], and the amount of evidence continues to increase [46]. The obtained results raise awareness of vitamin D supplementation in COVID-19; however, further clinical trials are required to produce detailed guidelines. As the effect of vitamin D supplementation in COVID-19 was shown to be modified by several factors, including the timing of therapy initiation [23], age [24], and comorbidity burden [26], we believe that prospective trials should be aimed at assessing the effectiveness of vitamin D therapy in vitamin D supplementation-naive patients and prioritize older and comorbid groups.

**Figure 3:**
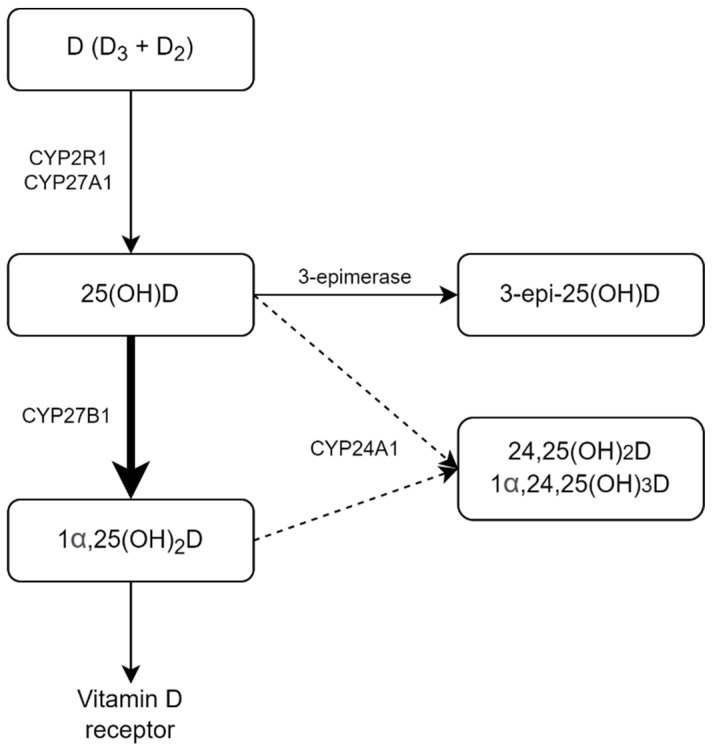
Schematic representation of the observed dysregulation of vitamin D metabolism in COVID-19 patients.

Looking deeper than the traditional 25OH-D_3_ levels, the first major finding was increased levels of active vitamin D metabolite (1,25(OH)_2_D_3_) in COVID-19 patients compared to the reference group, with a recovery observed alongside the course of the disease. The majority of COVID-19 patients were of older age, had higher creatinine levels and BMI compared to the reference group, and the association with a decrease — instead of an increase — of circulating active vitamin D metabolite was shown for these conditions [47,48,49]; hence one cannot explain the observed difference from this standpoint. The observed higher levels of 1,25(OH)_2_D_3_ cannot be fully explained by the lower activity of 24-hydroxylase due to the vitamin D deficiency either, since the production of 24,25(OH)_2_D_3_ appeared to be stable during the follow-up, unlike the levels of 1,25(OH)_2_D_3_. We can hypothesize that an increase in extrarenal 1α-hydroxylase activity might be a contributing factor and hereby question the generally accepted speculation that in the setting of vitamin D deficiency, lesser quantities of 25OH-D are available for the synthesis of 1,25(OH)_2_D, which might cause impairment of the innate immune response [50,51]. Given that, the localized intracrine mechanism is now considered a cornerstone of the interaction between vitamin D and the immune system [52]. Apart from that, previously reported studies on patients with sepsis showed a decrease in circulating 1,25(OH)_2_D_3_ levels [53] and the correlation of low 1,25(OH)_2_D_3_ levels with poor survival [54], so the observed findings may be specific to COVID-19. The proposed mechanism of vitamin D dysregulation among COVID-19 patients is illustrated in Figure 3.

The second significant finding was an increase in DBP levels and a concordant decrease in free 25OH-D in patients with COVID-19 in the acute phase of the disease, which is partially consistent with recent literature data [55] and indicates the presence of acute-phase protein properties in DBP. This feature has been described previously in various acute conditions, in particular, sepsis [56] and minor traumas [57].

Hypocalcemia in critically ill patients is universally known [58], and a high prevalence of low serum calcium levels are also described in hospitalized patients with COVID-19 [59]. The observed increase in circulating active vitamin D metabolite could be partly aimed at preventing hypocalcemia in COVID-19 patients with low total and free 25OH-D levels. Recent data on impaired compensatory PTH response in hypocalcemic patients with COVID-19, obtained by other research groups [60,61], support this hypothesis and may be due to negative feedback from 1,25(OH)_2_D_3_, although in our cohort, PTH levels were similar to the reference group.

Our study had several major limitations. First of all, individuals from the reference group were matched by the level of 25OH-D_3_, which allowed us to separate the effect of underlying vitamin D deficiency [32,37], but significantly differed in age, sex ratio, and BMI, and therefore it is not possible to separate the contribution of these factors within the framework of this work, although the authors consider such an impact as unlikely, taking into account the above-discussed data on the influence of these factors [47,48,49]. Also, the reference group was much smaller than the patients’ group, which requires a very cautious interpretation of the data obtained. Recruiting a matched control group was complicated due to the pandemic restrictions, especially for the elderly and comorbid individuals. Secondly, our study only allows us to make an assumption about the altered activity of vitamin D metabolism enzymes as the surrogate parameters were investigated and direct activity was not actually measured, as well as additional factors involved in the regulation of vitamin D metabolism (for instance, fibroblast growth factor-23) were lacking. This seems particularly important given that knowledge of the enzymes involved in vitamin D metabolism has increased in recent years. For example, other hydroxylases besides CYP2R1 (such as CYP3A4, CYP27A1, CYP2C11, and CYP2J2/3) have been shown to have a 25-hydroxylase activity that might affect 25OH-D_3_ levels [2], while CYP2C11 and CYP3A4 demonstrated 24-hydroxylase activity [62,63]; however, current literature data suggest that CYP24A1 remains the only established 24-hydroxylase involved in vitamin D metabolism [64]. The dietary habits of the patients were not taken into account, so that the low phosphorus dietary intake could have some contribution to the increase in 1,25(OH)_2_D_3_ production in COVID-19 patients [47]. Finally, the reduced amount of follow-up data attenuates the results of this part of the study. However, the study had a number of strengths: the substantial number of patients included, the wide range of parameters assessed for vitamin D metabolism and the high-quality method validated by an external control scheme used for measurement of vitamin D metabolites.

## 4. Materials and Methods

### 4.1. Study Population and Design

This was a retrospective cohort study based on a single-center experience. The main cohort included 119 adult patients hospitalized at the COVID-19 patient care hospital based in Moscow, Russia, for inpatient treatment during the period from 5 May to 4 June 2020. Diagnosis and inpatient treatment of COVID-19 patients was performed according to temporary federal guidelines [65]. COVID-19 was diagnosed with at least one of the following features in the presence of clinical findings suggestive of COVID-19: positive PCR nasal swab, positive PCR throat swab, a characteristic pattern of changes on chest CT. The severe course of the disease was defined within the framework of the current study as at least one of the following features: prescription of immunobiological treatment, either admission or transfer to the intensive care unit, fatal outcome. Recruitment of patients was carried out throughout the entire period of operation of the COVID-19 patient care hospital in a continuous manner, except for patients with insufficient residual serum for the planned laboratory measurements.

To exclude the direct impact of vitamin D deficiency on vitamin D metabolism, a reference group with similar to the COVID-19 patients’ serum 25OH-D_3_ levels were recruited. The reference group was comprised of 44 otherwise healthy adult individuals from the hospital staff who did not receive vitamin D supplementation for at least 3 months preceding the participation in the study.

**Figure 4:**
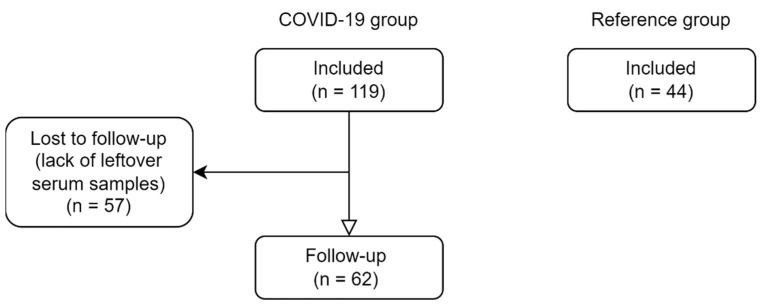
Schematic illustration of the study design.

The assessment included serum vitamin D metabolites (25OH-D_3_, 25OH-D_2_, 1,25(OH)_2_D_3_, 3-epi-25OH-D_3_, 24,25(OH)_2_D_3_ and D_3_), biochemical parameters (total calcium, albumin, phosphorus, creatinine), parathyroid hormone (PTH), vitamin D-binding protein (DBP) and free 25OH-D. Follow-up reassessment of COVID-19 patients further in the course of inpatient treatment included vitamin D metabolites, DBP, and free 25OH-D (Figure 4).

Anthropometric and clinical data were collected from the patients’ records.

The study was approved by the Ethics Committee of the Endocrinology Research Centre, Moscow, Russia, on 30 April 2020 (abstract of record No. 6). All patients signed informed consent to participate in the study.

### 4.2. Laboratory Measurements

After the completion of all routine laboratory testing, residual serum was stored at −80 °C until batched analysis, avoiding repeated freeze-thaw cycles. Biochemical parameters of blood serum were evaluated using the ARCHITECT c8000 analyzer (Abbott, Chicago, IL, USA) according to the standard methods using reagents from the same manufacturer. PTH levels were assessed by the electrochemiluminescence immunoassay (ELECSYS, Roche, Basel, Switzerland). Serum DBP and free 25OH-D levels were measured by enzyme-linked immunosorbent assay (ELISA) using commercial kits (Immundiagnostik AG, Bensheim, Germany, and DIAsource, ImmunoAssays S.A., Louvain-la-Neuve, Belgium respectively). The assay used for DBP levels assessment (Immundiagnostik AG, Bensheim, Germany) has a 3.3% intra- and inter-assay coefficient of variation (CV), the limit of blank is equal 0.154 mg/L, and the limit of detection is equal 0.944 mg/L. The assay used for free 25(OH)D levels assessment (DIAsource, ImmunoAssays S.A., Belgium) has 4.9% intra-assay CV and 5.9 inter-assay CV; the limit of blank is equal to 1.5 pg/mL and the limit of detection is equal 2.4 pg/mL. The limit of blank is defined as the highest measurement result that is likely to be observed (with a stated probability) for a blank sample, the limit of detection is defined as the lowest amount of analyte in a sample which can be detected but not necessarily quantitated as an exact value.

The serum levels of vitamin D metabolites (25OH-D_3_, 25OH-D_2_, 1,25(OH)_2_D_3_, 3-epi-25OH-D_3_, 24,25(OH)_2_D_3,_ and D_3_) were determined by ultra-high performance liquid chromatography in combination with tandem mass spectrometry (UPLC-MS/MS) using an in-house developed method, described earlier [66]. With this technique, the laboratory participates in the DEQAS quality assurance program (lab code 2388) and the results fall within the target range for the analysis of 25OH-D and 1,25(OH)_2_D metabolites in human serum (Supporting Information, Figures S1 and S2).

Albumin-adjusted serum calcium levels were calculated using the formula [67] total plasma calcium (mmol/L) = measured total plasma calcium (mmol/L) + 0.02 × (40 − measured plasma albumin (g/L)).

### 4.3. Statistical Analysis

Statistical analysis was performed using Statistica version 10.0 (StatSoft, Tulsa, OK, USA). All data were analyzed with non-parametric statistics and expressed as median [interquartile range], absolute values (percentages), or ratios. Mann–Whitney U-test and Fisher’s exact two-tailed test were used for comparisons between the two groups. Wilcoxon test was performed to evaluate changes in indices during follow-up. A *p*-value of less than 0.05 was considered statistically significant.

## 5. Conclusions

Taking into account all the limitations, this is the first study to our knowledge aimed at the comprehensive evaluation of vitamin D metabolism in COVID-19. Our data suggest that hospitalized patients with an acute course of COVID-19 have profound abnormalities in the metabolism of vitamin D apart from very low levels of 25OH-D (in particular, an increase in circulating active vitamin D metabolite and an increase in DBP levels) regardless of the clinical course of the disease. These alterations might likely exacerbate existing vitamin D deficiency and its negative impact.

## Figures and Tables

**Table 1:** General characteristics of the patients and individuals from the reference group. For a detailed description of the data format, please refer to Section 4.3.

Parameter	COVID-19 Group (n = 119)	Reference Group (n = 44)	*p*-Value
Age, years	61 [47; 73]	26 [24; 31]	<0.001
Sex (male/female), n	61/58	12/32	0.008
Body mass index, kg/m^2^	28.9 [24.9; 32.8]	21.5 [19.6; 25.7]	<0.001

**Table 2:** The course of the disease in COVID-19 patients.

Parameter	Value
*Evaluation at the time of admission*	
Time from symptom onset to hospitalization, days	9 [6; 11]
No. (%) of PCR positive	53 (45%)
No. (%) CT positive	115 (97%)
NEWS, points	4 [2; 6]
Body temperature, °C	37.4 [36.6; 38.0]
Respiratory rate, per minute	22 [20; 26]
Systolic blood pressure, mmHg	130 [119; 140]
Diastolic blood pressure, mmHg	80 [72; 90]
Lung involvement, %	28 [14; 42]
No. (%) of requiring respiratory support	54 (45%)
SpO2, %	94 [92; 96]
C-reactive protein, mg/L	75.5 [31.8; 139.6]
D-dimer, ng/mL	282 [164; 463]
Prothrombin time, s	12.2 [11.5; 13.2]
*Inpatient setting*	
Bed-days, n	12 [10; 15]
No. (%) of receiving antibiotic treatment	96 (81%)
No. (%) of receiving anticoagulant treatment	88 (74%)
No. (%) of treated with hydroxychloroquine	36 (30%)
No. (%) of treated with immunobiological drugs	21 (18%)
No. (%) of transferred to intensive care unit	11 (9%)
No. (%) of fatal outcomes	10 (8%)

Abbreviations: PCR, polymerase chain reaction; CT, computed tomography; NEWS, National Early Warning Score.

**Table 3:** Levels of the biochemical parameters and parathyroid hormone (PTH) in COVID-19 patients at the time of admission and in the reference group.

Laboratory Parameter	COVID-19 Group (n = 119)	Reference Group (n = 44)	Normal Range	*p*-Value
Creatinine, μmol/L	121 [88; 148]	70 [65; 78]	63–110 (male) 50–98 (female)	<0.001
Total calcium, mmol/L	2.19 [2.12; 2.30]	2.40 [2.34; 2.47]	2.15–2.55	<0.001
Albumin, g/L	39.5 [36; 42]	47 [46; 49]	35–50	<0.001
Albumin-adjusted calcium, mmol/L	2.23 [2.17; 2.28]	2.25 [2.20; 2.31]	2.15–2.55	0.06
Phosphorus, mmol/L	1.07 [0.93; 1.21]	1.14 [1.02; 1.26]	0.74–1.52	0.04
PTH, pg/mL	45.7 [29.8; 67.6]	40.8 [32.2; 52.2]	15–65	0.65

**Table 4:** Levels of free 25OH-D, vitamin D-binding protein (DBP), and vitamin D metabolites in COVID-19 patients and reference group.

Laboratory Parameter	COVID-19 Group, Admission (n = 119)	COVID-19 Group, Follow-Up (n = 62)	Reference Group (n = 44)	Normal Range	*p*-Value (Mann-Whitney) ^1^	*p*-Value (Wilcoxon)
Free 25OH-D, pg/mL	<LoB	2.5 [2.0; 4.7]	3.9 [3.2; 4.4]	2.4–35 ^2^	<0.001; 0.004	0.002
DBP, mg/L	450 [386; 515]	348 [283; 449]	392 [311; 433]	200–550 ^2^	<0.001; 0.13	<0.001
25OH-D_3_, ng/mL	10.8 [6.2; 15.6]	9.4 [5.2; 13.0]	10.9 [8.4; 13.1]	>30 ^3^	0.88; 0.18	<0.001
3-epi-25OH-D_3_, ng/mL	0.6 [0.4; 1.0]	0.5 [0.2; 0.8]	0.6 [0.4; 0.8]	not available	0.91; 0.48	<0.001
24,25(OH)_2_D_3_, ng/mL	0.4 [0.1; 0.8]	0.3 [0.1; 0.8]	0.5 [0.3; 0.9]	0.5–5.6 ^4^	0.07; 0.02	<0.001
1,25(OH)_2_D_3_, pg/mL	61 [44; 81]	33 [21; 45]	40 [35; 50]	25–66 ^4^	<0.001; 0.004	<0.001
25OH-D_3_/ 24,25(OH)_2_D_3_	25.9 [19.0; 46.2]	28.6 [19.0; 52.2]	18.6 [14.6; 34.7]	7–23 ^4^	0.001; <0.001	0.07
25OH-D_3_/ 1,25(OH)_2_D_3_	175 [112; 260]	279 [165; 449]	272 [200; 433]	not available	<0.001; <0.001	<0.001

^1^ The first value corresponds to the comparison of COVID-19 patients on presentation and the reference group and the second value corresponds to the comparison of COVID-19 patients during the follow-up examination and the reference group. ^2^ Ranges are specified according to kit manufacturers’ recommendations. ^3^ Range is given for a total of 25OH-D according to the clinical guidelines [40,41]; the 25OH-D_2_ fraction is negligible for the purposes of this study. ^4^ Ranges are given according to the literature data [42,43]. LoB—limit of blank.

**Table 5:** Comparison of subgroups of COVID-19 patients depending on the severity of the disease.

Laboratory Parameter	Moderately Severe (n = 85) ^1^	Severe (n = 34) ^1^	Normal Range	*p*-Value (Mann–Whitney) ^1^
Creatinine, μmol/L	124 [83; 151]	114 [101; 143]	63–110 (male) 50–98 (female)	0.68
Total calcium, mmol/L	2.20 [2.14; 2.31]	2.15 [2.04; 2.23]	2.15–2.55	0.04
Albumin, g/L	40 [36; 42]	39 [36; 42]	35–50	0.28
Albumin-adjusted calcium, mmol/L	2.24 [2.17; 2.29]	2.21 [2.15; 2.26]	2.15–2.55	0.16
Phosphorus, mmol/L	1.07 [0.94; 1.19]	1.09 [0.90; 1.25]	0.74–1.52	0.63
Free 25OH-D, pg/mL	<LoB 2.7 [2.1; 3.3]	<LoB 2.2 [1.8; 4.7]	2.4–35 ^2^	0.80 0.90
DBP, mg/L	450 [391; 509] 342 [290; 434]	442 [355; 531] 353 [267; 473]	200–550 ^2^	0.91 0.75
25OH-D_3_, ng/mL	10.9 [6.4; 16] 9.8 [5.7; 14.1]	8.8 [5.7; 15.5] 9.1 [3.3; 12.4]	>30 ^3^	0.29 0.48
3-epi-25OH-D_3_, ng/mL	0.6 [0.4; 1.1] 0.6 [0.4; 1.0]	0.7 [0.3; 0.8] 0.6 [0.2; 0.8]	not available	0.44 0.59
24,25(OH)_2_D_3_, ng/mL	0.5 [0.1; 0.9] 0.4 [0.1; 0.9]	0.3 [0.2; 0.7] 0.3 [0.1; 0.7]	0.5–5.6 ^4^	0.26 0.33
1,25(OH)_2_D_3_, pg/mL	64 [46; 83] 34 [24; 43]	54 [41; 79] 32 [15; 50]	25–66 ^4^	0.18 0.41

^1^ The first value corresponds to the baseline examination; the second value corresponds to the follow-up examination. ^2^ Ranges are specified according to kit manufacturers’ recommendations. ^3^ Range is given for a total of 25OH-D according to the clinical guidelines [40,41]; the 25OH-D_2_ fraction is negligible for the purposes of this study. ^4^ Ranges are given according to the literature data [42,43].

## Data Availability

Data is contained within the article or supplementary material.

## Supplementary Materials

The following supporting information can be downloaded at: https://www.mdpi.com/article/10.3390/ph15080906/s1, Figure S1: Comparison between DEQAS data for 25(OH)D scheme and our lab results, Figure S2: Comparison between DEQAS data for 1,25(OH)_2_D scheme and our lab results.

## References

[1] Guo Y.-R., Cao Q.-D., Hong Z.-S., Tan Y.Y., Chen S.-D., Jin H.-D., Tan K.-S., Wang D.-Y., Yan Y. (2020). The origin, transmission and clinical therapies on coronavirus disease 2019 (COVID-19) outbreak—An update on the status. Mil. Med. Res..

[2] Bikle D.D. (2014). Vitamin D metabolism, mechanism of action, and clinical applications. Chem. Biol..

[3] Hansdottir S., Monick M.M. (2011). Vitamin D Effects on Lung Immunity and Respiratory Diseases. Vitam. Horm..

[4] Bilezikian J.P., Bikle D., Hewison M., Lazaretti-castro M., Formenti A.M. (2020). Vitamin D and COVID-19. Eur. J. Endocrinol..

[5] Chun R.F., Liu P.T., Modlin R.L., Adams J.S., Hewison M. (2014). Impact of vitamin D on immune function: Lessons learned from genome-wide analysis. Front. Physiol..

[6] Liu N., Sun J., Wang X., Zhang T., Zhao M., Li H. (2021). Low vitamin D status is associated with coronavirus disease 2019 outcomes: A systematic review and meta-analysis. Int. J. Infect. Dis..

[7] Teshome A., Adane A., Girma B., Mekonnen Z.A. (2021). The Impact of Vitamin D Level on COVID-19 Infection: Systematic Review and Meta-Analysis. Front. Public Health.

[8] Kaya M.O., Pamukçu E., Yakar B. (2021). The role of vitamin D deficiency on the Covid-19: A systematic review and meta-analysis of observational studies. Epidemiol. Health.

[9] Szarpak L., Rafique Z., Gasecka A., Chirico F., Gawel W., Hernik J., Kaminska H., Filipiak K.J., Jaguszewski M.J., Szarpak L. (2021). A systematic review and meta-analysis of effect of vitamin D levels on the incidence of COVID-19. Cardiol. J..

[10] Akbar M.R., Wibowo A., Pranata R., Setiabudiawan B. (2021). Low Serum 25-hydroxyvitamin D (Vitamin D) Level Is Associated With Susceptibility to COVID-19, Severity, and Mortality: A Systematic Review and Meta-Analysis. Front. Nutr..

[11] Petrelli F., Luciani A., Perego G., Dognini G., Luigi P., Ghidini A. (2021). Therapeutic and prognostic role of vitamin D for COVID-19 infection: A systematic review and meta-analysis of 43 observational studies. J. Steroid Biochem. Mol. Biol..

[12] Margarucci L.M., Montanari E., Gianfranceschi G., Caprara C., Valeriani F., Piccolella A., Lombardi V., Scaramucci E., Spica V.R. (2021). The role of vitamin D in prevention of COVID-19 and its severity: An umbrella review. Acta Biomed..

[13] Chiodini I., Gatti D., Soranna D., Merlotti D., Mingiano C., Fassio A., Falchetti A., Eller-Vainicher C., Rossini M., Persani L. (2021). Vitamin D Status and SARS-CoV-2 Infection and COVID-19 Clinical Outcomes. Front. Public Health.

[14] Oscanoa T.J., Amado J., Vidal X., Laird E., Ghashut R.A., Romero-Ortuno R. (2021). The relationship between the severity and mortality of SARS-COV-2 infection and 25-hydroxy vitamin D concentration–a metaanalysis. Adv. Respir. Med..

[15] Wang Z., Joshi A., Leopold K., Jackson S., Christensen S., Nayfeh T., Mohammed K., Creo A., Tebben P., Kumar S. (2021). Association of vitamin D deficiency with COVID-19 infection severity: Systematic review and meta-analysis. Clin. Endocrinol..

[16] Bassatne A., Basbous M., Chakhtoura M., Zein O.E., Rahme M., Fuleihan G.E.-H. (2021). The link between COVID-19 and VItamin D (VIVID): A systematic review and meta-analysis. Metab. Clin. Exp..

[17] Chen J., Mei K., Xie L., Yuan P., Ma J., Yu P., Zhu W., Zheng C., Liu X. (2021). Low vitamin D levels do not aggravate COVID-19 risk or death, and vitamin D supplementation does not improve outcomes in hospitalized patients with COVID-19: A meta-analysis and GRADE assessment of cohort studies and RCTs. Nutr. J..

[18] Szarpak L., Filipiak K.J., Gasecka A., Gawel W., Koziel D., Jaguszewski M.J., Chmielewski J., Gozhenko A., Bielski K., Wroblewski P. (2021). Vitamin D supplementation to treat SARS-CoV-2 positive patients. Evidence from meta-analysis. Cardiol. J..

[19] Shah K., Saxena D., Mavalankar D. (2021). Vitamin D supplementation, COVID-19 and disease severity: A meta-analysis. QJM.

[20] Rocha A.P.d., Atallah A.N., Aldrighi J.M., Pires A.L.R., Puga M.E.d.S., Pinto A.C.P.N. (2021). Insufficient evidence for vitamin D use in COVID-19: A rapid systematic review. Int. J. Clin. Pract..

[21] Rawat D., Roy A., Maitra S., Shankar V., Khanna P., Baidya D.K. (2021). Vitamin D supplementation and COVID-19 treatment: A systematic review and meta-analysis. Diabetes Metab. Syndr. Clin. Res. Rev..

[22] Stroehlein J.K., Wallqvist J., Iannizzi C., Mikolajewska A., Metzendorf M.-I., Benstoem C., Meybohm P., Becker M., Skoetz N., Stegemann M. (2021). Vitamin D supplementation for the treatment of COVID-19: A living systematic review. Cochrane Database Syst. Rev..

[23] Pal R., Banerjee M., Bhadada S.K., Shetty A.J., Singh B., Vyas A. (2022). Vitamin D supplementation and clinical outcomes in COVID-19: A systematic review and meta-analysis. J. Endocrinol. Investig..

[24] Dramé M., Cofais C., Hentzien M., Proye E., Coulibaly P.S., Demoustier-Tampère D., Destailleur M.-H., Lotin M., Cantagrit E., Cebille A. (2021). Relation between vitamin D and COVID-19 in aged people: A systematic review. Nutrients.

[25] Hosseini B., Abd A.E., Ducharme F. (2022). Effects of Vitamin D Supplementation on COVID-19 Related Outcomes: A Systematic Review and Meta-Analysis. Nutrients.

[26] Giannini S., Passeri G., Tripepi G., Sella S., Fusaro M., Arcidiacono G., Torres M.O., Michielin A., Prandini T., Baffa V. (2021). Effectiveness of in-hospital cholecalciferol use on clinical outcomes in comorbid COVID-19 patients: A hypothesis-generating study. Nutrients.

[27] Azer S.M., Vaughan L.E., Tebben P.J., Sas D.J. (2021). 24-Hydroxylase Deficiency Due to CYP24A1 Sequence Variants: Comparison with Other Vitamin D−mediated Hypercalcemia Disorders. J. Endocr. Soc..

[28] Schlingmann K.P., Kaufmann M., Weber S., Lrwin A., Goos C., John U., Misselwitz J., Klaus G., Fehrenbach H., Wingin A.M. (2011). Mutations in CYP24A1 and Idiopathic Infantile Hypercalcemia. N. Engl. J. Med. Orig..

[29] Kaufmann M., Gallagher J.C., Peacock M., Schlingmann K.-P., Konrad M., DeLuca H.F., Sigueiro R., Lopez B., Mourino A., Maestro M. (2014). Clinical utility of simultaneous quantitation of 25-hydroxyvitamin D and 24,25-dihydroxyvitamin D by LC-MS/MS involving derivatization with DMEQ-TAD. J. Clin. Endocrinol. Metab..

[30] Kaufmann M., Morse N., Molloy B.J., Cooper D.P., Schlingmann K.P., Molin A., Kottler M.L., Gallagher J.C., Armas L., Jones G. (2017). Improved Screening Test for Idiopathic Infantile Hypercalcemia Confirms Residual Levels of Serum 24,25-(OH)_2_D_3_ in Affected Patients. J. Bone Miner. Res..

[31] Kaufmann M., Schlingmann K.P., Berezin L., Molin A., Sheftel J., Vig M., Gallagher J.C., Nagata A., Masoud S.S., Sakamoto R. (2021). Differential diagnosis of vitamin D–related hypercalcemia using serum vitamin D metabolite profiling. J. Bone Miner. Res..

[32] Cavalier E., Huyghebaert L., Rousselle O., Bekaert A.-C., Kovacs S., Vranken L., Peeters S., Goff C.L., Ladang A. (2020). Simultaneous measurement of 25(OH)-vitamin D and 24,25(OH)_2_-vitamin D to define cut-offs for CYP24A1 mutation and vitamin D deficiency in a population of 1200 young subjects. Clin. Chem. Lab. Med..

[33] Yu S., Wang D., Yin Y., Cheng Q., Xie S., Yu J., Sun D., Cheng X., Qiu L. (2019). Sources of variation evaluation of 24,25(OH)_2_D levels and the ratio of 25OHD to 24,25(OH)_2_D in apparently healthy Chinese adults: A multicenter cross-sectional study. J. Steroid Biochem. Mol. Biol..

[34] Francic V., Ursem S.R., Dirks N.F., Keppel M.H., Theiler-Schwetz V., Trummer C., Pandis M., Borzan V., Grübler M.R., Verheyen N.D. (2019). The effect of vitamin D supplementation on its metabolism and the vitamin D metabolite ratio. Nutrients.

[35] Ginsberg C., Katz R., de Boer I.H., Kestenbaum B.R., Chonchol M., Shlipak M.G., Sarnak M.J., Hoofnagle A.N., Rifkin D.E., Garimella P.S. (2018). The 24,25 to 25-hydroxyvitamin D ratio and fracture risk in older adults: The cardiovascular health study. Bone.

[36] Ginsberg C., Hoofnagle A.N., Katz R., Hughes-Austin J., Miller L.M., Becker J.O., Kritchevsky S.B., Shlipak M.G., Sarnak M.J., Ix J.H. (2021). The Vitamin D Metabolite Ratio Is Associated With Changes in Bone Density and Fracture Risk in Older Adults. J. Bone Miner. Res..

[37] Tang J.C.Y., Jackson S., Walsh N.P., Greeves J., Franser W.D., Facilityteam B. (2019). The dynamic relationships between the active and catabolic vitamin D metabolites, their ratios, and associations with PTH. Sci. Rep..

[38] Zelzer S., Prüller F., Curcic P., Sloup Z., Holter M., Herrmann M., Mangge H. (2021). Vitamin D metabolites and clinical outcome in hospitalized COVID-19 patients. Nutrients.

[39] Gallelli L., Mannino G.C., Luciani F., Sire A.d., Mancuso E., Gangemi P., Cosco L., Monea G., Averta C., Minchella P. (2021). Vitamin d serum levels in subjects tested for SARS-CoV-2: What are the differences among acute, healed, and negative COVID-19 patients? a multicenter real-practice study. Nutrients.

[40] Holick M.F., Binkley N.C., Bischoff-Ferrari H.A., Gordon M.C., Hanley D.A., Heaney R.P., Murad M.H., Weaver C.M. (2011). Evaluation, treatment, and prevention of vitamin D deficiency: An Endocrine Society clinical practice guideline. J. Clin. Endocrinol. Metab..

[41] Pigarova E.A., Rozhinskaya L.Y., Belaya J.E., Dzeranova L.K., Karonova T., Llyin A.V., Melnichenko G.A., Dedov I.I. (2016). Russian Association of Endocrinologists recommendations for diagnosis, treatment and prevention of vitamin D deficiency in adults. Probl. Endocrinol..

[42] Dirks N.F., Martens F., Vanderschueren D., Billen J., Pauwels S., Ackermans M.T., Endert E., Heijer M.d., Blankenstein M.A., Heijboer A.C. (2016). Determination of human reference values for serum total 1,25-dihydroxyvitamin D using an extensively validated 2D ID-UPLC–MS/MS method. J. Steroid Biochem. Mol. Biol..

[43] Tang J.C.Y., Nicholls H., Piec I., Washbourne C.J., Dutton J.J., Jackson S., Greeves J., Franser W.D. (2017). Reference intervals for serum 24,25-dihydroxyvitamin D and the ratio with 25-hydroxyvitamin D established using a newly developed LC–MS/MS method. J. Nutr. Biochem..

[44] Suplotova L., Avdeeva V., Pigarova E., Rozhinskaya L., Karonova T., Troshina E. (2021). The first Russian multicenter non-interventional registry study to study the incidence of vitamin D deficiency and insufficiency in Russian Federation. Ter. Arkh..

[45] Mercola J., Grant W.B., Wagner C.L. (2020). Evidence regarding vitamin D and risk of COVID-19 and its severity. Nutrients.

[46] Hernández J.L., Nan D., Fernandez-Ayala M., García-Unzueta M., Hernández-Hernández M.A., López-Hoyos M., Muñoz-Cacho P., Olmos J.M., Gutiérrez-Cuadra M., Ruiz-Cubillán J.J. (2020). Vitamin D Status in Hospitalized Patients with SARS-CoV-2 Infection. J. Clin. Endocrinol. Metab..

[47] Portale A.A., Halloran B.P., Morris R.C., Lonergan E.T. (1996). Effect of aging on the metabolism of phosphorus and 1,25-dihydroxyvitamin D in healthy men. Am. J. Physiol.–Endocrinol. Metab..

[48] Jean G., Souberbielle J.C., Chazot C. (2017). Vitamin D in chronic kidney disease and dialysis patients. Nutrients.

[49] Parikh S.J., Edelman M., Uwaifo G.I., Freedman R.J., Semega-Janneh M., Reynolds J., Yanovski J.A. (2004). The relationship between obesity and serum 1,25-dihydroxy vitamin D concentrations in healthy adults. J. Clin. Endocrinol. Metab..

[50] Mulligan J.K., Pasquini W.N., Carroll W.W., Williamson T., Reaves N., Patel K.J., Mappus E., Schlosser R.J., Atkinson C. (2017). Dietary vitamin D3 deficiency exacerbates sinonasal inflammation and alters local 25(OH)D_3_ metabolism. PLoS ONE.

[51] Vijayendra Chary A., Hemalatha R., Seshacharyulu M., Vasudeva Murali M., Jayaprakash D., Dinesh Kumar B. (2015). Vitamin D deficiency in pregnant women impairs regulatory T cell function. J. Steroid Biochem. Mol. Biol..

[52] Hewison M. (2010). Vitamin D and the intracrinology of innate immunity. Mol. Cell Endocrinol..

[53] Li C.H., Tang X., Wasnik S., Wang X., Zhang J., Xu Y., Lau K.-H.W., Nguyen H.B., Baylink D.J. (2019). Mechanistic study of the cause of decreased blood 1,25-Dihydroxyvitamin D in sepsis. BMC Infect. Dis..

[54] Nguyen H.B., Eshete B., Lau K.H.W., Sai A., Villarin M., Baylink D. (2013). Serum 1,25-Dihydroxyvitamin D: An Outcome Prognosticator in Human Sepsis. PLoS ONE.

[55] Subramanian S., Rhodes J.M., Taylor J.M., Milan A.M., Lane S., Hewison M., Chun R.F., Jorgensen A., Richardson P., Nitchingham D. (2022). Vitamin D, vitamin D–binding protein, free vitamin D and COVID-19 mortality in hospitalized patients. Am. J. Clin. Nutr..

[56] Dahl B., Schiødt F.V., Ott P., Fank W., William L., Jody B., Grant O. (2003). Plasma concentration of Gc-globulin is associated with organ dysfunction and sepsis after injury. Crit. Care Med..

[57] Dahl B., Schiødt F.V., Rudolph S., Ott P., Kiær T., Heslet L. (2001). Trauma stimulates the synthesis of Gc-globulin. Intensive Care Med..

[58] Kelly A., Levine M.A. (2013). Hypocalcemia in the critically ill patient. J. Intensive Care Med..

[59] Filippo L.d., Formenti A.M., Rovere-Querini P., Carlucci M., Conte C., Ciceri F., Zangrillo A., Giustina A. (2020). Hypocalcemia is highly prevalent and predicts hospitalization in patients with COVID-19. Endocrine.

[60] Filippo L.D., Allora A., Locatelli M., Querini P.R., Frara S., Banfi G., Giustina A. (2021). Hypocalcemia in COVID-19 is associated with low vitamin D levels and impaired compensatory PTH response. Endocrine.

[61] Hashemipour S., Kiani S., Shahsavari P., Afshar S., Ghobadi A., Khairkhahan S.M.R.H., Badri M., Farzam S.S., Sohrabi H., Seddighi M. (2022). Hypocalcemia in hospitalized patients with COVID-19: Roles of hypovitaminosis D and functional hypoparathyroidism. J. Bone Miner. Metab..

[62] Roizen J.D., Li D., O’Lear L., Javaid M.K., Shaw N.J., Ebeling P.R., Nguyen H.H., Rodda C.P., Thummel K.E., Thacher T.D. (2018). CYP3A4 mutation causes Vitamin D-dependent rickets type 3. J. Clin. Investig..

[63] Rahmaniyan M., Patrick K., Bell N.H. (2005). Characterization of recombinant CYP2C11: A vitamin D 25-hydroxylase and 24-hydroxylase. Am. J. Physiol.-Endocrinol. Metab..

[64] Tuckey R.C., Cheng C.Y.S., Slominski A.T. (2019). The serum vitamin D metabolome: What we know and what is still to discover. J. Steroid Biochem. Mol. Biol..

[65] Temporary guidelines – prevention, diagnosis and treatment of new coronavirus infection (COVID-19) Ministry of Health of Russia. Version VI. 28.04.2020. https://static-1.rosminzdrav.ru/system/attachments/attaches/000/050/116/original/28042020_MR_COVID-19_v6.pdf.

[66] Povaliaeva A., Pigarova E., Zhukov A., Bogdanov V., Dzeranova L., Mel’nikova O., Pekareva E., Malysheva N., Ioutsi V., Nikankina L. (2020). Evaluation of vitamin D metabolism in patients with type 1 diabetes mellitus in the setting of cholecalciferol treatment. Nutrients.

[67] Thode J., Juul-Jørgensen B., Bhatia H.M., Kjaerulf-Nielsen M., Bartels P.D., Fogh-Andersen N., Siggaard-Andersen O. (1989). Comparison of serum total calcium, albumin-corrected total calcium, and ionized calcium in 1213 patients with suspected calcium disorders. Scand. J. Clin. Lab. Investig..

